# Executive Functioning as a Mediator Between Digital Media Exposure and Communication Outcomes in Children and Adolescents

**DOI:** 10.3390/jintelligence14040067

**Published:** 2026-04-17

**Authors:** Csongor Toth, Brigitte Osser, Laura Ioana Bondar, Gyongyi Osser, Roland Fazakas, Nicoleta Anamaria Pascalau, Ramona Nicoleta Suciu, Liliana-Oana Pobirci, Corina Dalia Toderescu, Bombonica Gabriela Dogaru

**Affiliations:** 1 Doctoral School of Biomedical Sciences, University of Oradea, 410087 Oradea, Romania; toth.csongor1@student.uoradea.ro (C.T.); bondar.lauraioana@student.uoradea.ro (L.I.B.); gabriela.dogaru@umfcluj.ro (B.G.D.); 2 Faculty of Physical Education and Sport, “Aurel Vlaicu” University of Arad, 310130 Arad, Romania; gyongyi.osser@uav.ro; 3 Department of Biology and Life Sciences, Faculty of Medicine, “Vasile Goldiș” Western University of Arad, 310025 Arad, Romania; fazakas.roland@uvvg.ro; 4 Multidisciplinary Doctoral School, “Vasile Goldiș” Western University of Arad, 310025 Arad, Romania; 5 Department of Psycho Neuroscience and Recovery, Faculty of Medicine and Pharmacy, University of Oradea, 410087 Oradea, Romania; nicoleta.pascalau@didactic.uoradea.ro (N.A.P.); opobirci@uoradea.ro (L.-O.P.); 6 Department of Pharmaceutical Sciences, Faculty of Pharmacy, “Vasile Goldiș” Western University of Arad, 310045 Arad, Romania; toderescu.corina@uvvg.ro; 7 Department of Medical Rehabilitation, “Iuliu Hațieganu” University of Medicine and Pharmacy Cluj-Napoca, 410087 Cluj-Napoca, Romania; 8 Clinical Rehabilitation Hospital, 400347 Cluj-Napoca, Romania

**Keywords:** children and adolescents, digital media use, executive functioning, language development, parental mediation, pragmatic communication, screen time, social communication, working memory

## Abstract

Background/Objectives: The increasing prevalence of digital media use among children and adolescents has raised concerns regarding its potential impact on cognitive and communication development. Previous research has linked higher screen exposure to poorer language outcomes; however, the mechanisms underlying these associations remain insufficiently understood, particularly with respect to pragmatic communication. The present study aimed to examine the relationships between daily screen time, executive functioning (EF), and communication-related outcomes, and to test whether EF mediates the association between digital media exposure and pragmatic communication and language performance. Methods: A cross-sectional observational study was conducted with 240 children and adolescents aged 6–15 years. Caregivers reported children’s daily screen time, digital consumption and communication skills. EF was assessed using performance-based tasks measuring inhibitory control, working memory, and cognitive flexibility. Language performance was evaluated using a standardized composite measure. Pearson correlations, mediation analyses with bootstrapped confidence intervals, and factorial analyses of variance were performed, controlling for age, sex, parental mediation, and educational content exposure. Results: Higher daily screen time was significantly associated with lower EF, weaker pragmatic communication, and poorer language performance. EF was positively related to both pragmatic and language outcomes and partially mediated the relationship between screen time and communication measures. Educational digital content and parental mediation showed positive associations with EF and communication outcomes, whereas recreational content exhibited negative associations. Group comparisons indicated that negative associations between screen exposure and developmental outcomes were more pronounced in younger children. Conclusions: These findings suggest that EF may represent a key intermediary mechanism underlying the association between digital media exposure and communication-related development. The results highlight the importance of considering not only the quantity but also the quality and context of children’s digital media use, particularly during early developmental stages.

## 1. Introduction

Digital technologies have become increasingly integrated into children’s daily lives, shaping how they learn, interact, and spend their time across home and educational settings. Screen-based devices such as smartphones, tablets, computers, and televisions are now deeply embedded in family routines, educational contexts, and leisure activities, resulting in unprecedented levels of daily digital media exposure from early childhood onward (Odgers & Jensen, 2020; Kar et al., 2025; Munzer et al., 2026; Borges et al., 2025; Fiore et al., 2025; Osser et al., 2025a). While digital media offer potential benefits for learning and access to information, growing concern has emerged regarding their implications for children’s cognitive, linguistic, and socio-communicative development (da Silva Junior et al., 2025; Misirli et al., 2025; Mallawaarachchi et al., 2024; Karani et al., 2022).

A substantial body of research has documented associations between higher levels of screen time and less favorable developmental outcomes in children, including reduced language abilities, weaker academic performance, and difficulties in attention and self-regulation (Li et al., 2025; Santos et al., 2022; Madigan et al., 2020). From a language development perspective, excessive screen exposure has been linked to smaller vocabularies, delayed expressive language, and poorer comprehension, particularly during early childhood (Rayce et al., 2024; Malik et al., 2025; Nwachukwu et al., 2025; Panjeti-Madan & Ranganathan, 2023). One proposed explanation is that screen use, under certain conditions, may displace experiences that are critical for language learning, such as face-to-face interaction, conversational turn-taking, and shared attention with caregivers and peers (Wan et al., 2025; Toledo-Vargas et al., 2025; Gillioz et al., 2025).

Notably, findings in this area are not entirely consistent, and a growing body of research has reported null or more nuanced associations between digital media use and cognitive outcomes. Some studies suggest that the relationship between screen use and executive functioning (EF) may be weak or non-significant when accounting for individual differences, patterns of use, and contextual factors. For instance, research using objective measures of smartphone engagement has found no consistent association between problematic smartphone use and EF after controlling for relevant covariates (Hartanto et al., 2023). These mixed findings highlight the complexity of digital media effects and underscore the importance of moving beyond simple duration-based models toward approaches that consider underlying mechanisms and contextual influences.

Beyond structural language skills, pragmatic communication—the ability to use language appropriately in social contexts—has received comparatively less attention in the digital media literature. Pragmatic skills rely heavily on social-cognitive processes, including perspective-taking, contextual sensitivity, and flexible language use (Misirli et al., 2025; Bambini & Lecce, 2025; Brushe et al., 2024). Emerging evidence suggests that high levels of screen time may be associated with weaker social communication abilities, including difficulties in conversational coherence and social responsiveness (Panjeti-Madan & Ranganathan, 2023; Gath et al., 2026; Patel et al., 2025). However, the mechanisms underlying these associations remain insufficiently understood.

EF refers to a group of higher-order cognitive processes—typically including inhibitory control, working memory, and cognitive flexibility—that support goal-directed behavior and adaptive functioning (Filipe et al., 2023; Andre & Maintenant, 2025; Choi et al., 2026; Thériault-Couture et al., 2025; Herreras, 2025). EF supports children’s ability to regulate attention, inhibit inappropriate responses, maintain conversational goals, and flexibly adapt language to social and contextual demands (Oshchepkova et al., 2025b; Howard et al., 2023; Q. Wang et al., 2025; Cano-Villagrasa et al., 2024). Strong EF has been consistently associated with better language performance and more effective pragmatic communication across childhood and adolescence (Howard et al., 2023; Udhnani et al., 2020; Huang & Li, 2025).

At the same time, several studies have reported negative associations between higher screen time and EF outcomes, particularly in younger children (Lakicevic et al., 2025; Fitzpatrick et al., 2025; Vohr et al., 2021; Mortimer et al., 2024). Theoretical accounts suggest that excessive or passive screen use may reduce opportunities for activities that naturally foster executive control, such as unstructured play, sustained problem-solving, and socially guided interaction (Clemente-Suárez et al., 2024; Putnick et al., 2023). Despite these parallel lines of evidence, relatively few studies have directly tested whether EF functions as a mechanistic link between digital media exposure and communication-related outcomes.

Additionally, the directionality of associations between digital media use, EF, and communication outcomes is likely to be complex and potentially bidirectional. While the present study is grounded in models proposing that higher screen exposure may be associated with weaker EF and, in turn, with less favorable communication outcomes, alternative pathways are also plausible. For example, children with lower EF or weaker self-regulation may be more likely to engage in higher levels of screen use, or caregivers may rely on digital media as a regulatory tool for children with greater attentional or behavioral difficulties. Given the cross-sectional design of the present study, causal inferences cannot be drawn; however, examining EF as a mediating mechanism provides a theoretically informed framework for understanding these associations.

Furthermore, digital media exposure is not a unitary construct. Increasing evidence indicates that the developmental impact of screen use depends not only on duration but also on content quality and contextual factors (Nuvoli et al., 2025; Xiao et al., 2025; Bal et al., 2024). Educational digital content has been associated with more positive cognitive and language outcomes, particularly when it is age-appropriate and actively engaging. In contrast, predominantly recreational or fast-paced content has been linked to weaker executive control and attentional difficulties (Nwachukwu et al., 2025; Langlais et al., 2025; Naik et al., 2025; Srisinghasongkram et al., 2025).

Parental mediation represents another critical contextual factor shaping children’s digital experiences. Active mediation strategies—such as setting limits, discussing content, and engaging in joint media use—have been associated with better self-regulation, language development, and more adaptive media habits (Z. Zhao et al., 2025; Nagata et al., 2025; Bayar et al., 2025). Conversely, low levels of caregiver involvement may exacerbate the potential negative effects of excessive screen exposure.

Developmental timing further complicates this picture. EF and communication skills undergo rapid maturation during childhood, with younger children generally exhibiting greater sensitivity to environmental influences. Several studies suggest that early childhood may represent a particularly vulnerable period in which high screen exposure is more strongly associated with negative developmental outcomes (Xiao et al., 2025; Takahashi et al., 2023; J. Zhao et al., 2022; Zhang et al., 2021). However, few studies have directly examined age-related differences in these associations within a single integrative framework.

Taken together, existing research highlights robust associations between digital media exposure, EF, and communication development, yet important gaps remain. In particular, there is a need for studies that integrate EF as a mediating mechanism, examine pragmatic communication alongside broader language outcomes, distinguish between content types and parental mediation, and explicitly consider developmental differences across childhood and adolescence.

This study addresses these gaps by examining associations between daily screen time, EF, and developmental outcomes (pragmatic communication and language performance) in a sample of children and adolescents aged 6 to 15 years. Specifically, the study investigates whether EF is statistically associated with the relationship between screen exposure and communication-related outcomes while accounting for content type, parental mediation, and age. In addition, group differences across screen-time categories and developmental stages are examined to explore age-related sensitivity to digital media exposure.

Based on the theoretical framework and prior empirical findings, the following hypotheses were formulated. First, higher daily screen time is expected to be negatively associated with EF, including inhibitory control, working memory, and cognitive flexibility, as well as with pragmatic communication and language performance. Second, EF is expected to be positively associated with communication outcomes and to mediate the relationship between screen time and developmental outcomes. Third, exposure to educational digital content and higher levels of parental mediation are expected to be positively associated with EF and communication outcomes, whereas recreational content is expected to show negative associations. Finally, age-related differences are anticipated, with older children demonstrating higher EF and communication performance, and with more pronounced differences across screen-time categories in younger age groups.

## 2. Materials and Methods

### 2.1. Study Design and Procedure

The present study used a cross-sectional, observational design to examine associations between daily digital media exposure, EF, and developmental outcomes (pragmatic communication and language performance) in children and adolescents. Data collection took place at Vinga Technological High School (Arad County, Romania) between December 2024 and December 2025.

Vinga Technological High School is a comprehensive educational institution that includes multiple levels of education, ranging from primary to secondary and vocational training. This structure allows for the inclusion of children across a broad age range (6–15 years) within a single school setting.

The selection of this school was based on practical and logistical considerations, including established collaboration with the institution and the feasibility of conducting standardized assessments across multiple age groups within a single setting. While this approach enabled efficient data collection and consistency in testing conditions, it may also introduce selection bias. In particular, the characteristics of families associated with this school (e.g., educational orientation, access to digital resources, and patterns of media use) may differ from those of the broader population. These factors should be considered when interpreting the generalizability of the findings beyond similar educational and sociocultural contexts.

Data collection was conducted during the academic year, with assessments scheduled in coordination with school staff to minimize disruption to classes. Children were assessed individually in a quiet room within the school, while caregivers completed questionnaires regarding children’s digital media use (daily screen time and content composition) and parental mediation practices. EF tasks and outcome measures were administered following standardized instructions by trained members of the research team.

### 2.2. Participants

Participants were 240 children and adolescents (121 girls, 119 boys) aged between 6 and 15 years (M = 10.6, SD = 2.7), recruited from Vinga Technological High School, Arad County, Romania. The sample included students enrolled in primary, lower secondary, and upper secondary educational levels.

To reflect key stages of cognitive, executive, and communicative development, participants were grouped into four developmental stages based on chronological age:

- G1: 6–7 years (*n* = 58);
- G2: 8–9 years (*n* = 60);
- G3: 10–12 years (*n* = 61);
- G4: 13–15 years (*n* = 61).

#### 2.2.1. Inclusion Criteria

Participants were included in the study if they met the following criteria:

- Were between 6 and 15 years of age;
- Were enrolled as students at the participating school at the time of data collection;
- Had no reported history of neurological disorders, intellectual disability, or uncorrected sensory impairments;
- Demonstrated sufficient proficiency in Romanian to complete the assessment tasks;
- Had written informed consent provided by a parent or legal guardian.

#### 2.2.2. Exclusion Criteria

Participants were excluded from the study if any of the following conditions applied:

- Caregivers reported a diagnosed neurodevelopmental disorder (e.g., autism spectrum disorder, attention-deficit/hyperactivity disorder);
- The child was unable to complete the assessment procedures due to fatigue, lack of cooperation, or noncompliance during testing;
- Written informed consent from a parent or legal guardian was not obtained.

**Figure 1:**
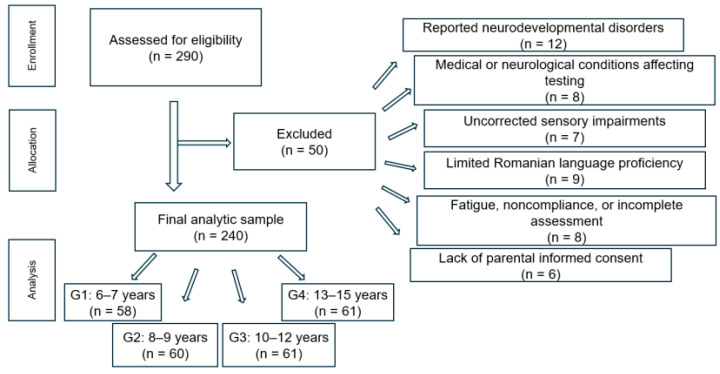
Flowchart of participant selection and inclusion.

A total of 290 students were initially assessed for eligibility. Of these, 50 participants were excluded based on predefined exclusion criteria, including reported neurodevelopmental disorders (*n* = 12), medical or neurological conditions affecting testing (*n* = 8), uncorrected sensory impairments (*n* = 7), limited Romanian language proficiency (*n* = 9), fatigue, noncompliance, or incomplete assessment data (*n* = 8), and lack of parental informed consent (*n* = 6). The final sample consisted of 240 children and adolescents who were included in all subsequent analyses (Figure 1).

### 2.3. Measures

#### 2.3.1. Screen Time and Digital Media Exposure

Daily screen time was assessed using a structured caregiver-report questionnaire designed to capture children’s typical digital media use on weekdays and weekends. The questionnaire consisted of 4 items assessing average daily screen exposure across different time periods (weekday use, weekend use, and device-specific prompts). Caregivers were asked to estimate the average number of minutes per day the child spent using screen-based devices, including televisions, computers, tablets, smartphones, and gaming consoles.

A weighted average daily screen-time score (min/day) was calculated by combining weekday and weekend estimates. Screen time was treated as a continuous variable in correlational and mediation analyses and categorized into three groups for group-comparison analyses: ≤1 h/day, 1–2 h/day, and >2 h/day.

Because this measure reflects estimated duration of behavior across distinct contexts rather than a latent psychological construct, internal consistency indices (e.g., Cronbach’s α) are not applicable. However, caregiver-report measures of children’s screen time have been widely used in developmental research and have demonstrated acceptable validity when compared with time-use diaries and objective tracking methods.

#### 2.3.2. Digital Content Composition

Caregivers also reported on the typical composition of the child’s digital media use by estimating the proportion of time spent on educational versus recreational content. Educational content was defined as media designed to support learning, cognitive development, or academic skills (e.g., educational videos, learning applications, instructional programs), whereas recreational content included entertainment-oriented media (e.g., cartoons, social media, video games, non-educational videos).

Caregivers estimated the proportion of time spent on educational and recreational content. These estimates were treated as continuous variables and did not necessarily sum to 100%, reflecting the approximate nature of caregiver reports. Although mean values approximate 100% due to averaging across participants, individual-level estimates were not constrained to sum to 100%. These variables were used as continuous predictors in correlational and mediation analyses and were included as covariates in regression models.

#### 2.3.3. Parental Mediation of Digital Media Usage

Parental mediation of digital media use was assessed using a structured caregiver-report scale adapted from commonly used parental mediation frameworks in the digital media literature (Valkenburg et al., 2013; Nikken & Jansz, 2014). The scale included 8 items assessing both active and restrictive mediation practices, such as setting rules for screen time, monitoring content, discussing digital media experiences, and engaging in joint media use.

Caregivers responded to each item on a 4-point Likert scale, ranging from 1 (low mediation) to 4 (high mediation). A composite parental mediation score was gathered by averaging responses across items, with higher scores indicating greater caregiver involvement. The scale demonstrated good internal consistency in the present sample (Cronbach’s α = 0.84).

#### 2.3.4. Assessment of EF

EF was assessed using age-appropriate performance-based tasks measuring three core domains: inhibitory control, working memory, and cognitive flexibility. Tasks were administered individually in a quiet school setting by trained researchers following standardized instructions.

- Inhibitory control (Go/No-Go task). Participants completed a child-adapted Go/No-Go task consisting of 80 trials, including 56 Go trials (70%) and 24 No-Go trials (30%). Stimuli were presented sequentially on a computer screen, and participants were instructed to respond as quickly as possible to Go stimuli while withholding responses to No-Go stimuli. Each stimulus was presented for 1000 ms with an inter-stimulus interval of 1000 ms. Performance was indexed using commission error rate (percentage of responses to No-Go trials), with higher error rates indicating poorer inhibitory control. Accuracy on Go trials and mean reaction time (RT) for correct Go responses were also recorded; however, the primary measure used in analyses was the No-Go commission error rate.
- Working memory (Digit Span). Working memory was assessed using forward and backward Digit Span tasks administered according to standard procedures. Sequences began with two digits (forward) and three digits (backward), increasing progressively in length. Two trials were administered at each sequence length. Testing was discontinued when the participant failed both trials at a given length. The total score was calculated as the total number of correctly recalled sequences across forward and backward conditions, with higher scores indicating better working memory performance.
- Cognitive flexibility was assessed using a child-adapted Dimensional Change Card Sort (DCCS) task. The task consisted of three phases: a pre-switch phase (sorting by an initial rule, e.g., color), a post-switch phase (sorting by a new rule, e.g., shape), and a mixed phase requiring flexible switching between rules. Each phase included 10 trials, for a total of 30 trials. Performance was indexed by accuracy (percentage of correct responses) in the post-switch and mixed phases, which are considered the most sensitive indicators of cognitive flexibility.

To create a composite EF score, task-specific performance indices were first aligned such that higher values reflected better performance (i.e., commission error rates were reverse-scored). Each measure was rescaled to a common metric (0–100 range) to ensure comparability across tasks with different scoring scales. The overall EF composite score was calculated by averaging the three task indices, with higher scores indicating better EF. Inter-task correlations ranged from r = 0.58 to 0.74, indicating moderate convergence across EF domains. No formal factor analysis was conducted; however, the composite was derived based on established theoretical models conceptualizing EF as comprising interrelated but distinct components. Domain-specific scores were also retained for correlational analyses.

#### 2.3.5. Pragmatic Communication

Pragmatic communication was assessed using the Children’s Communication Checklist-2 (CCC-2), a standardized caregiver-report measure designed to evaluate children’s use of language in social contexts (Bishop, 2013). The Romanian-adapted version of the CCC-2 was used in the present study. The instrument consists of 70 items organized into multiple subscales assessing pragmatic aspects of communication, including conversational turn-taking, use of context-appropriate language, understanding of implicit meanings, and maintenance of topic coherence.

Caregivers rated each item on a Likert-type scale according to the frequency of observed behaviors. A total pragmatic communication score was computed following standardized scoring procedures, with higher scores indicating stronger pragmatic language skills. In the present sample, the CCC-2 demonstrated high internal consistency (Cronbach’s α = 0.91). The instrument has been widely used and has demonstrated good reliability and validity across diverse populations.

#### 2.3.6. Language Performance

General language ability was assessed using a standardized composite measure of receptive and expressive language skills derived from age-appropriate language assessments tasks administered as part of the assessment protocol. These tasks were informed by commonly used clinical language frameworks (Clinical Evaluation of Language Fundamentals) and included measures of vocabulary, sentence comprehension, and expressive language abilities.

Raw scores from each component were transformed to a common metric based on sample distributions and adjusted for age to account for developmental differences. The language composite score was calculated by averaging performance across tasks, with higher scores indicating better overall language ability.

Although the composite reflects domains commonly assessed in standardized language instruments, it does not represent a single standardized test score with population norms. Therefore, findings should be interpreted as reflecting relative performance within the sample rather than normative clinical classification. The composite score was used as a primary outcome variable in correlational, mediation, and group-comparison analyses.

#### 2.3.7. Covariates

Age (in years) and sex were included as demographic covariates in all mediation models. In addition, parental mediation and the shared amount of educational digital content were included as covariates to account for contextual influences on digital media use and developmental outcomes.

### 2.4. Procedure

Data collection was conducted at Vinga Technological High School, Arad County, Romania, between December 2024 and December 2025, during the academic year. All assessments were carried out in the school setting in rooms designated by the school administration to ensure a quiet and distraction-free environment.

Prior to data collection, parents or legal guardians received detailed written information about the study objectives, procedures, and confidentiality safeguards. Written informed consent was obtained from all caregivers, and verbal assent was obtained from participating children and adolescents before the assessment sessions.

Performance-based EF and language assessments were administered individually by trained researchers following standardized instructions. Each assessment session lasted approximately 45–60 min, depending on the participant’s age and pace. Short breaks were offered as needed, particularly for younger children, to minimize fatigue and maintain attention. Testing was discontinued if a participant showed signs of excessive fatigue, distress, or unwillingness to continue.

Caregiver-report questionnaires assessing screen time, digital consumption, parental influence, and communication were completed independently by parents or legal guardians. Questionnaires were distributed either in paper format or electronically, according to caregiver preference, and were returned to the research team within one week.

All participants were assigned unique identification codes, and data were anonymized prior to analysis. Only aggregated data were used for statistical analyses.

### 2.5. Statistical Analysis

Statistical analyses were conducted using IBM SPSS Statistics (Version 26.0; IBM Corp., Armonk, NY, USA). Descriptive statistics (means, standard deviations, and ranges) were computed for all study variables to characterize the sample and assess data distribution.

Pearson correlation analyses were performed to examine bivariate associations among daily screen time, digital content composition, parental mediation, EF measures, pragmatic communication, and language performance.

To test the proposed mediation hypotheses, mediation analyses were conducted using ordinary least squares regression with bootstrapped confidence intervals (5000 resamples), implemented in the PROCESS macro for SPSS (Model 4). Daily screen time served as the independent variable (X), EF (EF composite score) as the mediator (M), and pragmatic communication and language composite scores as outcome variables (Y). Age, sex, parental mediation, and the proportion of educational digital content were included as covariates in all mediation models. Indirect effects were considered statistically significant when the bootstrapped 95% confidence interval did not include zero.

Group differences were examined using analyses of variance (ANOVAs), with age group (four levels) and daily screen-time category (≤1 h/day, 1–2 h/day, >2 h/day) entered as between-subject factors. EF, pragmatic communication, and language composite scores were used as dependent variables. Effect sizes were reported as partial eta squared (ηp^2^).

Statistical significance was set at *p* < 0.05 for all analyses.

### 2.6. Ethical Considerations

The study was approved by the Institutional Ethics Committee of Vinga Technological High School, Arad County, Romania (protocol code 1435/2, approved on 16 October 2023). All procedures were conducted in accordance with the ethical principles outlined in the Declaration of Helsinki.

Participation was voluntary, and caregivers and participants were informed of their right to withdraw from the study at any time without penalty. Written informed consent was obtained from parents or legal guardians, and verbal assent was obtained from all participating children and adolescents prior to participation.

Participant confidentiality and data protection were strictly maintained. Personal identifying information was stored separately from research data, and all datasets used for analysis were fully anonymized. Data were accessible only to the research team and were used exclusively for research purposes.

## 3. Results

The section presents descriptive statistics, correlational analyses, mediation models, and group comparisons examining the associations between digital media exposure, EF, and developmental outcomes in children and adolescents.

### 3.1. Descriptive Characteristics of the Sample

A total of *n* = 240 children and adolescents were included in the final analytic sample after applying the inclusion and exclusion criteria. Participants ranged in age from 6 to 15 years (M = 10.6, SD = 2.7). The sex distribution was approximately balanced, with 121 girls (50.4%) and 119 boys (49.6%). The distribution of participants across developmental stages and sex is presented in Table 1.

Participants were grouped in four developmental stages based on age: G1: 6–7 years (*n* = 58), G2: 8–9 years (*n* = 60), G3: 10–12 years (*n* = 61), and G4: 13–15 years (*n* = 61).

Daily digital media exposure showed substantial variability across participants (min–max = 20–360 min/day), with a mean screen time of M = 154.8 min/day (SD = 61.3). Consistent with developmental trends, average screen exposure tended to be higher in older age groups. To support categorical comparisons, participants were classified into three screen-time categories (≤1 h/day, 1–2 h/day, and >2 h/day), with the largest proportion observed in the >2 h/day category.

Participants were distributed across screen-time categories as follows: ≤1 h/day (*n* = 46), 1–2 h/day (*n* = 72), and >2 h/day (*n* = 122). A detailed breakdown of participants across age group × screen-time categories is presented in Table 2.

Descriptive statistics for screen exposure variables, digital content composition, parental mediation, EF measures, and outcome variables are summarized in Table 3. The average proportion of educational content was M = 34.1% (SD = 17.6), while recreational content accounted for M = 65.9% (SD = 17.6). Parental mediation scores ranged from 1.0 to 4.0, with a mean of M = 2.43 (SD = 0.84), indicating moderate levels of caregiver involvement in guiding children’s digital activities.

EF performance demonstrated adequate variability and broad score ranges across participants. The overall EF composite score had a mean of M = 52.8 (SD = 9.6, min–max = 28–74). Among EF domains, inhibitory control (M = 54.1, SD = 10.2, min–max = 30–78), working memory (M = 51.7, SD = 9.1, min–max = 29–72), and cognitive flexibility (M = 52.6, SD = 9.4, min–max = 31–75) showed progressive increases across developmental stages, consistent with expected maturational effects.

Outcome measures, including pragmatic communication and the language composite score, also demonstrated appropriate dispersion (pragmatic communication: min–max = 18–48; language composite: min–max = 32–88), supporting their suitability for correlational, group-comparison, and mediation analyses.

### 3.2. Correlations Among Study Variables

Correlation analyses were conducted in order to examine associations between screen time and digital content, parental mediation, EF measures, and outcome variables. As shown in Table 4, daily screen time was moderately and negatively correlated with overall EF (r = −0.32, *p* < 0.001), as well as with its core components, including inhibitory control (r = −0.29, *p* < 0.001), working memory (r = −0.27, *p* < 0.001), and cognitive flexibility (r = −0.25, *p* < 0.001).

EF showed moderate-to-strong positive associations with outcome measures. The EF composite score was positively correlated with pragmatic communication (r = 0.46, *p* < 0.001) and with the language composite score (r = 0.49, *p* < 0.001). Similar patterns were observed for individual EF domains, with inhibitory control and working memory demonstrating the strongest associations with pragmatic and linguistic performance.

Screen time was also negatively associated with pragmatic communication (r = −0.28, *p* < 0.001) and the language composite score (r = −0.31, *p* < 0.001). Educational digital content showed small but significant positive correlations with EF (r = 0.21, *p* < 0.01) and outcome measures (r range = 0.18–0.23, *p* < 0.01), whereas recreational content exhibited the opposite pattern, showing negative correlations with EF and developmental outcomes.

Parental mediation was positively correlated with EF (r = 0.30, *p* < 0.001), pragmatic communication (r = 0.33, *p* < 0.001), and language performance (r = 0.29, *p* < 0.001), and was inversely related to daily screen time (r = −0.35, *p* < 0.001). Age was positively associated with EF and outcome variables, reflecting expected developmental progression.

Overall, the correlation pattern supports the proposed mediation model in which EF represents a plausible intermediary mechanism linking digital media exposure to linguistic and pragmatic outcomes.

### 3.3. Mediation Analysis: EF as a Mediator

Mediation analyses were conducted to test the role of EF in the association between digital media exposure and developmental outcomes. Ordinary least squares regression with bootstrapped confidence intervals (5000 resamples) was applied, with daily screen time as the independent variable (X), EF (EF composite score) as the mediator (M), and pragmatic communication and language composite score as outcome variables (Y). Age, sex, parental mediation, and the proportion of educational digital content were included as covariates in all models. These findings should be interpreted as reflecting statistical associations consistent with a mediation model; however, given the cross-sectional design, causal relationships among variables cannot be established.

#### 3.3.1. Mediation Model for Pragmatic Communication

As shown in Table 5, daily screen time significantly predicted EF (path a), with higher screen exposure associated with lower EF performance (β = −0.32, *p* < 0.001). EF, in turn, significantly predicted pragmatic communication after controlling for screen time and covariates (path b; β = 0.41, *p* < 0.001).

The total effect of screen time on pragmatic communication (path c) was statistically significant (β = −0.28, *p* < 0.001). When EF was included in the model, the direct effect of screen time on pragmatic communication (path c′) was reduced but remained statistically significant (β = −0.15, *p* = 0.012), indicating partial mediation.

The indirect effect of screen time on pragmatic communication through EF was statistically significant (β = −0.13), with a bootstrapped 95% confidence interval that did not include zero (−0.19 to −0.08; Table 6).

#### 3.3.2. Mediation Model for Language Composite Score

A similar mediation pattern was observed for the language composite outcome. Screen time significantly predicted EF (path a; β = −0.32, *p* < 0.001), and EF significantly predicted language performance after controlling for screen time and covariates (path b; β = 0.44, *p* < 0.001).

The total effect of screen time on the language composite score was statistically significant (path c; β = −0.31, *p* < 0.001). After inclusion of EF, the direct effect of screen time on language performance was attenuated but remained significant (path c′; β = −0.17, *p* = 0.008), again supporting partial mediation.

The indirect effect of screen time on language performance via EF was statistically significant (β = −0.14), with a bootstrapped 95% confidence interval ranging from −0.21 to −0.09 (Table 6).

### 3.4. Group Differences in EF and Developmental Outcomes

To further examine differences in EF and developmental outcomes across age and screen exposure levels, a series of analyses of variance (ANOVAs) were conducted. Age group (G1: 6–7 years; G2: 8–9 years; G3: 10–12 years; G4: 13–15 years) and daily screen-time category (≤1 h/day, 1–2 h/day, >2 h/day) were included as between-subject factors. EF (EF composite score), pragmatic communication, and language composite score served as dependent variables, with results summarized in Table 7.

#### 3.4.1. EF Across Age and Screen-Time Groups

The analysis revealed a significant main effect of age group on EF (F(3, 228) = 18.42, *p* < 0.001, ηp^2^ = 0.20), indicating progressively higher EF performance in older age groups. A significant main effect of screen-time category was also observed (F(2, 228) = 9.76, *p* < 0.001, ηp^2^ = 0.08), with children in the ≤1 h/day category demonstrating the highest EF scores and those in the >2 h/day category demonstrating the lowest.

In addition, the age group × screen-time category interaction was statistically significant (F(6, 228) = 2.31, *p* = 0.036, ηp^2^ = 0.06). Post hoc comparisons indicated that differences in EF across screen-time categories were more pronounced in younger children (G1 and G2), whereas EF scores in older age groups (G3 and G4) were less strongly differentiated by screen exposure level.

#### 3.4.2. Pragmatic Communication and Language Performance

Similar patterns were observed for pragmatic communication and language outcomes. For pragmatic communication, significant main effects of age group (F(3, 228) = 15.27, *p* < 0.001, ηp^2^ = 0.17) and screen-time category (F(2, 228) = 7.84, *p* < 0.001, ηp^2^ = 0.06) were found. Children with lower daily screen exposure showed higher pragmatic communication scores across age groups. The interaction between age group and screen-time category for pragmatic communication was not statistically significant (F(6, 228) = 1.98, *p* = 0.071, ηp^2^ = 0.05).

For the language composite score, both age group (F(3, 228) = 21.03, *p* < 0.001, ηp^2^ = 0.22) and screen-time category (F(2, 228) = 10.18, *p* < 0.001, ηp^2^ = 0.08) yielded significant main effects. The interaction between age group and screen-time category for the language composite score was not statistically significant (F(6, 228) = 2.05, *p* = 0.061, ηp^2^ = 0.05).

Overall, these findings indicate that higher screen exposure is associated with lower EF and communication-related outcomes across age groups, supporting the inclusion of age as a covariate in subsequent mediation analyses.

## 4. Discussion

The present study investigated the associations between digital media exposure, EF, and developmental outcomes in children and adolescents, with a particular focus on pragmatic communication and language performance. Building on prior research and extending existing models, this study examined not only direct relationships between screen time and developmental outcomes, but also the mediating role of EF and the influence of age, content type, and parental mediation. Overall, the findings provide converging evidence that EF may represent a key intermediary mechanism underlying the association between digital media exposure and communication-related development.

### 4.1. Screen Time, EF, and Developmental Outcomes

Daily screen time was negatively associated with overall EF as well as with its core components—namely inhibitory control, working memory, and cognitive flexibility. These findings are consistent with a growing body of research indicating that higher levels of digital media exposure are associated with reduced self-regulatory capacity, attentional control, and goal-directed behavior in children and adolescents (Lakicevic et al., 2025; Maeneja et al., 2025; Caamaño-Navarrete et al., 2025; Muppalla et al., 2023; Osser et al., 2025b). From a developmental perspective, excessive screen use may limit opportunities for activities that naturally support EF, such as unstructured play, sustained problem-solving, and socially guided interaction (Torres et al., 2025; Cheng et al., 2025; T. Zhao et al., 2026; Yaffe et al., 2025).

The observed effect sizes were in the small-to-moderate range, suggesting that screen time is not a deterministic factor but rather a meaningful environmental influence within a broader constellation of developmental inputs. This pattern aligns with prior work emphasizing that digital media exposure contributes incrementally—rather than predominantly—to variability in EF outcomes (Kar et al., 2025; Muppalla et al., 2023; Oh et al., 2023).

Higher daily screen exposure was also associated with poorer pragmatic communication and language performance. This finding supports previous research showing that increased screen use may displace experiences critical for language development, including face-to-face interaction, conversational turn-taking, and shared attention with caregivers and peers. Importantly, the associations observed in the present study extended beyond structural language skills, encompassing pragmatic aspects of communication that rely heavily on social-cognitive processing and executive control (Rayce et al., 2024; Brushe et al., 2024; Massaroni et al., 2024; Mustonen et al., 2022).

It is important to note that screen exposure represents a heterogeneous construct, and its developmental implications are likely influenced by contextual factors such as content type, interactivity, and caregiver involvement. Moreover, group comparison analyses indicated that negative associations between screen exposure and EF were more pronounced in younger children, suggesting heightened vulnerability during earlier developmental stages when executive systems are still emerging (Lakicevic et al., 2025; Xiao et al., 2025; Ma et al., 2025). Taken together, these findings underscore the importance of considering both the quantity and developmental timing of screen exposure when evaluating its potential impact on cognitive and communication-related outcomes.

### 4.2. EF as a Mediating Mechanism

A central contribution of the present study lies in demonstrating that EF partially mediates the relationship between daily screen time and both pragmatic communication and language performance. In line with the proposed hypotheses, higher screen exposure was associated with lower EF, which in turn predicted poorer communication-related outcomes. This mediation pattern is consistent with theoretical models suggesting that EF serves as a core mechanism through which environmental factors influence higher-order developmental skills (Bal et al., 2024; Shokrkon & Nicoladis, 2022; Scionti et al., 2023; Bustamante et al., 2023).

The finding of partial rather than full mediation is particularly informative. While EF accounted for a substantial proportion of the association between screen time and developmental outcomes, screen exposure retained a direct effect on pragmatic and language performance even after controlling for EF and relevant covariates. This suggests that screen time may influence communication development through multiple pathways, including—but not limited to—executive control processes (Malik et al., 2025; Wan et al., 2025; Massaroni et al., 2024).

From a developmental standpoint, EF is known to support language and pragmatic skills by enabling children to maintain conversational goals, inhibit irrelevant responses, flexibly adapt to contextual cues, and integrate linguistic input over time. The strong associations observed between EF and both outcome measures in the present study are consistent with prior work highlighting the role of inhibitory control and working memory in discourse management and social communication (Oshchepkova et al., 2025a; Bambini et al., 2021).

The present mediation findings extend existing literature by empirically linking digital media exposure to communication outcomes through EF within a single integrative model. Rather than treating EF as a parallel correlate, the results indicate that variations in cognitive control capacity help explain why higher screen exposure is associated with less favorable developmental outcomes. This mechanistic interpretation suggests that EF may be a relevant target for preventive and intervention-oriented approaches aimed at supporting children’s communication development within contemporary media contexts (Olive et al., 2024; Qu et al., 2025). Importantly, these findings reflect statistical associations consistent with a mediation model and should not be interpreted as evidence of causal relationships, given the cross-sectional design.

### 4.3. Role of Digital Content and Parental Mediation

Beyond the amount of daily screen exposure, the present findings highlight the importance of digital content characteristics and parental mediation in shaping developmental outcomes. In line with the proposed hypotheses, exposure to educational digital content was positively associated with EF and communication-related outcomes, whereas recreational content showed the opposite pattern. These results support the growing consensus that screen time is not a unitary construct and that the developmental implications of digital media use depend substantially on content quality and cognitive demands (Fan et al., 2022; Wu et al., 2026; Selak et al., 2025).

Educational digital content may support EF and language development by promoting active engagement, sustained attention, and structured learning experiences. Such content often incorporates explicit linguistic input, problem-solving elements, and goal-oriented tasks, which may place greater demands on executive control processes. In contrast, recreational content—particularly fast-paced or purely entertainment-oriented material—may provide fewer opportunities for sustained cognitive engagement and self-regulation (Maeneja et al., 2025; Namazi & Sadeghi, 2024; Hinten et al., 2025). The present findings are consistent with prior research suggesting that educational content may buffer some of the negative associations between overall screen exposure and developmental outcomes.

Parental mediation emerged as another important contextual factor. Higher levels of caregiver involvement were associated with better EF, stronger pragmatic communication, and improved language performance, as well as with lower overall screen time. These findings align with previous studies indicating that parental mediation can enhance the quality of children’s digital experiences by guiding content selection, encouraging reflection, and supporting joint engagement (Koch et al., 2025; Bukhalenkova et al., 2023; Guellai et al., 2022; McArthur et al., 2022).

Importantly, parental mediation may operate through multiple mechanisms. Active mediation strategies—such as discussing content, asking questions, and linking digital experiences to real-world contexts—may directly support language and EF. At the same time, parental regulation of screen time may help preserve opportunities for non-digital activities that are critical for cognitive and communicative development, including social interaction and play (Wijaya et al., 2025; Tan et al., 2025; J. Wang et al., 2024; Lin et al., 2025).

In the present study, parental mediation was operationalized as a composite construct combining both active and restrictive strategies. However, recent research suggests that these forms of mediation may have distinct and, in some cases, divergent associations with children’s developmental outcomes. Active mediation—such as discussion, co-use, and guided engagement with digital content—has been more consistently linked to positive cognitive and communicative outcomes, whereas restrictive mediation—focused on rule-setting and time limits—may show more variable effects depending on developmental stage and context (Commodari et al., 2024; Görgülü & Özer, 2023; Liu et al., 2023). Future research should therefore differentiate between mediation styles to better understand how specific parental practices interact with children’s media use and executive and communication outcomes.

Taken together, these findings underscore the need for a nuanced understanding of digital media exposure that extends beyond screen duration alone. The results suggest that content quality and caregiver involvement can meaningfully shape the relationship between screen use, EF, and developmental outcomes, reinforcing the importance of family context in children’s engagement with digital media.

### 4.4. Developmental Differences and Age-Related Effects

Developmental stage emerged as a critical factor in understanding the associations between digital media exposure, EF, and communication-related outcomes. Across analyses, age was positively associated with EF and language and pragmatic performance, reflecting well-established maturational trajectories in cognitive control and communication skills (Tervo-Clemmens et al., 2023; Goudas et al., 2024; Michel et al., 2025). These findings confirm that EF and communication abilities continue to develop throughout childhood and adolescence, providing an essential context for interpreting the effects of screen exposure.

Importantly, group comparison analyses indicated that the negative association between screen time and EF was more pronounced in younger children than in older age groups. Specifically, children in early developmental stages who were exposed to higher levels of daily screen time showed comparatively lower EF scores, whereas this differentiation was attenuated in adolescents. This pattern is consistent with theoretical models positing that younger children may be more vulnerable to environmental influences due to their ongoing development and reduced stability of their executive control systems (Vohr et al., 2021; Zhang et al., 2021; McHarg et al., 2020; Onyeaka et al., 2022).

A similar age-related trend was observed for pragmatic communication and language performance. Although higher screen exposure was associated with poorer outcomes across age groups, the strength of this association tended to be greater in younger children. This finding aligns with prior research suggesting that early childhood represents a particularly sensitive period for language and social-communication development, during which, reduced exposure to interactive linguistic input may have more pronounced consequences (da Silva Junior et al., 2025; Takahashi et al., 2023; T. C. Zhao et al., 2022).

These developmental differences may be explained by several mechanisms. Younger children rely more heavily on direct social interaction and external scaffolding to support EF and language learning, whereas older children and adolescents possess more mature cognitive control systems that may buffer some of the potential negative effects of screen exposure. Additionally, older individuals may engage with digital media in more goal-directed or educational ways, further moderating its developmental impact (Morgan et al., 2021; Baker et al., 2025; Meredith & Silvers, 2024; Pöpplau et al., 2024).

Overall, the present findings emphasize that the effects of digital media exposure cannot be fully understood without considering developmental timing. The heightened sensitivity observed in younger children underscores the importance of age-appropriate media guidelines and early preventive strategies aimed at supporting EF and communication development during critical periods of growth.

### 4.5. Implications for Research, Practice, and Policy

The present findings have several important implications for future research, clinical and educational practice, and the development of evidence-based policy regarding children’s digital media use. By identifying EF as a key intermediary mechanism linking screen exposure to communication-related outcomes, this study contributes to a more nuanced understanding of how digital environments interact with cognitive and developmental processes.

From a research perspective, the results underscore the importance of moving beyond simple associations between screen time and developmental outcomes. Future studies should continue to adopt mechanism-focused approaches, examining how EF and other cognitive processes mediate the effects of digital media exposure on language, social communication, and broader developmental domains. Longitudinal designs will be particularly valuable in clarifying developmental trajectories and the directionality of these relationships.

In addition, the present findings highlight the need for more fine-grained assessments of digital media use. Distinguishing between content types, levels of interactivity, and patterns of co-use with caregivers may help explain variability in outcomes across individuals and developmental stages. Future research should also explore domain-specific EF as parallel mediators and examine potential moderated mediation effects involving age, parental mediation, and socioeconomic context.

The findings have direct relevance for practitioners working with children and families, including educators, psychologists, speech-language therapists, and pediatric professionals. Rather than focusing exclusively on limiting screen time, interventions may benefit from emphasizing content quality, developmentally appropriate media use, and active caregiver involvement. Supporting EF—through structured play, goal-directed activities, and scaffolding strategies—may represent a promising pathway for mitigating potential negative effects of excessive screen exposure on communication development.

For clinical populations or children exhibiting language or self-regulation difficulties, careful consideration of digital media habits may be particularly important. Practitioners can play a key role in guiding families toward balanced media use that complements, rather than replaces, rich social and communicative experiences.

At the policy level, the present findings support recommendations that move beyond universal screen-time limits toward more context-sensitive guidelines. Policies and public health messages should account for developmental stage, content type, and family context, recognizing that younger children may be more vulnerable to high levels of screen exposure. Emphasizing parental mediation and the promotion of high-quality educational content may enhance the effectiveness of media guidelines aimed at supporting healthy development.

Taken together, these implications suggest that effective responses to the challenges posed by digital media exposure require coordinated efforts across research, practice, and policy domains. By integrating insights from EF research with media-use frameworks, stakeholders can better support children’s cognitive and communicative development in today’s media-saturated developmental landscape.

### 4.6. Limitations and Future Directions

Several limitations of the present study should be acknowledged when interpreting the findings. First, the cross-sectional design precludes causal inferences regarding the relationships among screen time, EF, and developmental outcomes. Although the mediation analyses were theoretically grounded and statistically robust, longitudinal designs are needed to clarify developmental directionality and to examine how changes in digital media exposure relate to trajectories of EF and communication skills over time.

Second, measurement of screen time, content composition, parental mediation relied on caregiver report, which may be subject to recall bias or social desirability effects. Importantly, these measurement limitations may have affected the accuracy of reported media use patterns and caregiver behaviors, potentially attenuating or inflating observed associations. In particular, caregiver reports may not fully capture the complexity, variability, or context of children’s actual digital media use. Therefore, the findings related to media exposure and parental mediation should be interpreted with caution. Future studies would benefit from incorporating objective or digitally logged measures of media use, as well as more fine-grained assessments distinguishing between specific platforms, levels of interactivity, and social contexts of use.

Third, although the sample covered a broad range, the study did not examine socioeconomic status, cultural context, or home literacy environment as moderators of the observed associations. These factors may influence both digital media practices and developmental outcomes and should be systematically integrated into future models to enhance ecological validity and generalizability.

Fourth, participants were recruited from a single educational institution, which may limit the generalizability of the findings. Although Vinga Technological High School is a comprehensive institution that includes multiple educational levels, it may not be fully representative of the broader population of Romanian children and adolescents. School-level selection factors—such as local socioeconomic characteristics, educational orientation, and access to digital resources—may have influenced patterns of screen time, content use, and parental mediation observed in the sample. Therefore, the findings should be interpreted with caution and may not generalize beyond similar educational and sociocultural contexts. Future research should aim to replicate these results in more diverse, multi-site, and socioeconomically heterogeneous samples.

Fifth, a formal prospective power analysis was not conducted prior to data collection. Although the sample size (*n* = 240) is comparable to those commonly used in mediation research and sufficient to detect indirect effects of small-to-moderate magnitude using bootstrapping procedures, future research should incorporate simulation-based or mediation-specific power analyses to more precisely estimate the sample sizes required to detect indirect effects.

Sixth, the study was not pre-registered, and the hypotheses were not formally registered prior to data collection. Although the hypotheses were theoretically derived, future research would benefit from pre-registration to enhance transparency and reduce potential biases in analytic decisions.

In addition, the present study focused on EF as a composite mediator. While this approach provided a coherent mechanism-level account, future research should further explore domain-specific EF—such as inhibitory control, working memory, and cognitive flexibility—within parallel or sequential mediation frameworks. Such analyses may offer more precise insights into which cognitive processes are most sensitive to digital media exposure and most predictive of communication outcomes.

Finally, future research should examine potential moderated mediation effects, testing whether the strength of indirect pathways varies as a function of age, parental mediation strategies, content type, or developmental risk status. Intervention-based and experimental designs targeting EF or media-use patterns would also be valuable in determining whether modifying these factors can lead to improvements in pragmatic communication and language development.

Despite these limitations, the present study contributes to the literature by integrating EF into a comprehensive model of digital media effects on developmental outcomes. By identifying both risk and protective factors, the findings provide a foundation for future longitudinal, mechanistic, and intervention-oriented research aimed at supporting children’s cognitive and communicative development in digital contexts.

## 5. Conclusions

The present study contributes to the growing literature on digital use and child development by demonstrating that EF appears to play a central role in the association between daily screen exposure, pragmatic communication and language outcomes in children and adolescents. Across analyses, higher screen time was consistently associated with lower EF and poorer communication-related performance, while stronger EF was linked to more favorable developmental outcomes.

Importantly, the mediation findings indicate that EF partially explains the relationship between screen exposure and language and pragmatic skills, suggesting that digital media may influence communication development indirectly by shaping underlying cognitive control processes. These results underscore the value of moving beyond duration-based models of screen use toward mechanistic frameworks that account for cognitive and developmental pathways.

The study also highlights the importance of contextual factors. Exposure to educational digital content and higher levels of parental mediation were associated with better EF and communication outcomes, whereas predominantly recreational content showed less favorable associations. In addition, developmental timing emerged as a key consideration, with younger children appearing more sensitive to higher levels of screen exposure than adolescents.

Taken together, these findings support a nuanced perspective on digital media use in childhood, emphasizing that both the quantity and quality of screen exposure—alongside cognitive capacities and family context—are critical for understanding developmental outcomes. By integrating EF into models of digital media effects, the present study provides a foundation for future longitudinal and intervention-oriented research aimed at promoting healthy cognitive and communicative development in contemporary childhood contexts.

## Figures and Tables

**Table 1:** Distribution of the Sample by Age Group and Sex (*n* = 240).

Age Group	Age Range (years)	Total (*n*)	Girls (*n*)	Boys (*n*)
G1	6–7	58	29	29
G2	8–9	60	31	29
G3	10–12	61	31	30
G4	13–15	61	30	31
Total	6–15	240	121	119

**Table 2:** Distribution of Participants across Age Group and Screen-Time Category (*n* = 240).

Age Group	Age Range (years)	≤1 h/day	1–2 h/day	>2 h/day	Total
G1	6–7	18	22	18	58
G2	8–9	14	20	26	60
G3	10–12	8	16	37	61
G4	13–15	6	14	41	61
Total	6–15	46	72	122	240

**Table 3:** Descriptive Statistics of Screen Use, EFs, and Outcome Variables.

Variable	M	SD	Min–Max
Screen time (min/day)	154.8	61.3	20–360
Educational content (%)	34.1	17.6	0–100
Recreational content (%)	65.9	17.6	0–100
Parental mediation	2.43	0.84	1.0–4.0
EF composite (task scores)	52.8	9.6	28–74
Inhibitory control (task scores)	54.1	10.2	30–78
Working memory (task scores)	51.7	9.1	29–72
Cognitive flexibility (task scores)	52.6	9.4	31–75
Pragmatic communication (scale score)	31.6	7.8	18–48
Language composite (scale score)	61.2	11.9	32–88

**Table 4:** Pearson Correlations among Screen Time, EF, and Outcome Variables (*n* = 240).

Variable	1	2	3	4	5	6	7	8	9	10
1. Screen time (min/day)	—									
2. Educational content (%)	−0.34 ***	—								
3. Recreational content (%)	0.42 ***	−0.61 ***	—							
4. Parental mediation	−0.35 ***	0.29 ***	−0.33 ***	—						
5. EF composite (task scores)	−0.32 ***	0.21 **	−0.26 ***	0.30 ***	—					
6. Inhibitory control	−0.29 ***	0.19 **	−0.23 ***	0.27 ***	0.74 ***	—				
7. Working memory	−0.27 ***	0.18 **	−0.21 **	0.25 ***	0.71 ***	0.62 ***	—			
8. Cognitive flexibility	−0.25 ***	0.17 *	−0.20 **	0.23 ***	0.69 ***	0.58 ***	0.60 ***	—		
9. Pragmatic communication	−0.28 ***	0.20 **	−0.24 ***	0.33 ***	0.46 ***	0.41 ***	0.43 ***	0.39 ***	—	
10. Language composite	−0.31 ***	0.23 ***	−0.27 ***	0.29 ***	0.49 ***	0.45 ***	0.47 ***	0.42 ***	0.61 ***	—

Note. * *p* < 0.05, ** *p* < 0.01, *** *p* < 0.001.

**Table 5:** Regression Coefficients for Mediation Models Predicting Pragmatic Communication and Language Performance.

Path	Predictor → Outcome	β	SE	*p*
a	Screen time → EF composite	−0.32	0.05	<0.001
b_1_	EF composite → Pragmatic communication	0.41	0.06	<0.001
c	Screen time → Pragmatic communication (total effect)	−0.28	0.05	<0.001
c′	Screen time → Pragmatic communication (direct effect)	−0.15	0.06	0.012
b_2_	EF composite → Language composite	0.44	0.05	<0.001
c	Screen time → Language composite (total effect)	−0.31	0.05	<0.001
c′	Screen time → Language composite (direct effect)	−0.17	0.06	0.008

Note. All models adjusted for age, sex, parental mediation, and proportion of educational digital content.

**Table 6:** Bootstrapped Indirect Effects of Screen Time on Developmental Outcomes via EF.

Outcome Variable	Indirect Effect (a × b)	Bootstrapped 95% CI
Pragmatic communication	−0.13	−0.19 to −0.08
Language composite	−0.14	−0.21 to −0.09

Note. Bootstrapped confidence intervals based on 5000 resamples. Indirect effects are considered statistically significant when the confidence interval does not include zero.

**Table 7:** Effects of Age Group and Screen-Time Category on EF and Developmental Outcomes.

Dependent Variable	Effect	F	*p*	ηp^2^
EF composite	Age group	18.42	<0.001	0.20
	Screen-time category	9.76	<0.001	0.08
	Age × Screen time	2.31	0.036	0.06
Pragmatic communication	Age group	15.27	<0.001	0.17
	Screen-time category	7.84	<0.001	0.06
	Age × Screen time	1.98	0.071	0.05
Language composite	Age group	21.03	<0.001	0.22
	Screen-time category	10.18	<0.001	0.08
	Age × Screen time	2.05	0.061	0.05

Note. Age group (4 levels) and screen-time category (≤1 h/day, 1–2 h/day, >2 h/day) were entered as between-subject factors. Effect sizes are reported as partial eta squared (ηp^2^).

## Data Availability

The data supporting the findings of this study are not publicly available due to ethical and privacy restrictions involving minors. Anonymized data may be made available from the corresponding author upon reasonable request.

## Abbreviations

The following abbreviations are used in this manuscript:

EF	Executive Functioning
CCC-2	Children’s Communication Checklist–2
DCCS	Dimensional Change Card Sort
